# Hospital Meetings, &c.

**Published:** 1898-03-05

**Authors:** 


### HOSPITAL MEETINGS, &C.

### ROYAL FREE HOSPITAL.

In the absence of Mr. Justice Bruce, Mr. Charles Burt presided over the seventieth court of governors of the Royal Free Hospital held at the hospital on Wednesday, February 23rd. In referring to the fact that the institution had reached its seventieth year, the Chairman remarked that instead of growing weaker it had grown stronger with age. During 1897 the total number of in-patients under treatment was 2,262, while the out-patients numbered 35,118, as compared with 38,992 in the previous year, the decrease being attributed to the care exercised to prevent any abuse of the charity. According to the report the weekly board are devoting considerable attention to the out-patient problem. An inquiry officer or almoner has been attached to the hospital for the past three years, and this official is instructed to refer applicants needing food rather than medicine to the Poor Law authorities, first medical relief, however, being given when required. The income for the year amounted to £10,138, including legacies to the extent of £3,663, while the expenditure amounted to £12,143. Three beds have been endowed during the last year, £1,000 having been received from Mr. H. L. Florence, and £2,000 from Miss Marter for that purpose. There was an increase in the expenditure of £270 in consequence of the decision to provide all the patients with tea, sugar, and butter, and the average weekly cost per bed, after deducting the expense of out-patients, amounted to £1 5s. 7d. At the close of 1897 163 students were attending the London School of Medicine for Women, with which the hospital is associated. During the year 36 new students entered the school, 31 of whom are taking a full course of study. Twenty students have become registered medical practitioners, 10 of them having taken the M.B. degree of the London University, while three medical women, formerly students of the school and hospital, have recently been appointed for plague duty in India. For some years past it has been recognised that certain structural alterations are necessary to bring the hospital up to the requirements of modern sanitary science. Unfortunately, however, the committee cannot sanction further outlay on improvements, and the rough estimates they have obtained show that the cost of the alterations most needed would be £8,650. It was reported that Mr. Burt, Mr. Sheppard, and Mr. Wilmott Evans had been appointed as representatives of the hospital on the Central Hospital Council for London, and that in order to strengthen the association of the medical staff with the general management of the hospital, Dr. Samuel West, the senior physician, and Mr. A. Boyce Barrow, the senior surgeon, have joined the Committee of Management. The Marquis of Northampton, Sir Edward Durning Lawrence, Sir J. Blundell Maple, M.P., and Sir Charles Hall, Q.C., M.P., having been appointed vice-presidents, Mrs. Garrett Anderson, M D., proposed a resolution expressing regret at the death of Sir James Stansfield, who for many years was a member of the Committee of Management. She said she was glad to be entrusted with that resolution because at a critical period in the history of the Medical School for Women Sir James arranged for the union with this hospital, which had proved of such great advantage. The resolution was carried unanimously.

### EAST LONDON HOSPITAL FOR CHILDREN.

Mr. H. W. Trinder presided over the annual Court of Governors held at the hospital on Wednesday, February 23rd. The attendance was small. The report shows that in 1897 1,616 in-patients were treated, being the largest number in the hospital's history. The out-patients, on the other hand, show a decrease from 34,941 in 1896, to 33,468 last year, this being principally due to the efforts made to prevent undue pressure on the medical staff by the continuous increase in the number of out-patients. The medical staff is the same as in 1891, when the out-patients numbered only 21,216, and it is therefore proposed to appoint a casualty officer, who will act as enquiry officer, and some addition to the staff may be necessary unless the out-patients can be reduced in number. Care is taken to exclude patients where the parents can afford to pay for the service of local practitioners. But in Shadwell such persons are few, and a recent examination showed that more than 50 per cent. of the children in the wards were the children of labourers. During 1897 measles and other contagious diseases were rife in East London, and in consequence of children developing such diseases in the hospital, wards had to be closed five times. The accounts show that both annual subscriptions and donations have increased, and the legacies amounted to £5,493, of which £4,137 has been invested by the Board. At the end of 1897 there was a balance of £400 to the good, so that the Jubilee year, which the Board had looked forward to with anxiety, proved eminently satisfactory from the financial standpoint. The Convalescent Home at Bognor is to be completed by September 30th, considerable progress having been made with the building during the recent favourable weather. For the completion of the Home a further £3,000 will be required. A new operating theatre is considered necessary at the hospital, and the Board have decided to incur the expense, which will probably amount to £800 or £1,000. They are also urged by the resident medical officer to provide a ward for whooping-cough cases, and they hope to deal with this question in their next report. The adoption of the report was seconded by Canon Akers, and after the General Committee had been elected the Board of Management, with the addition of the Rev. G. Harris, rector of St. George's-in-the-East, was reappointed. It is desired to enlist the sympathy of ladies with the work of the institution, and with this object an association of ladies is to be formed which will meet from time to time at the hospital.

### THE MIDDLESEX HOSPITAL.

The annual report of the board of the Middlesex Hospital was presented to a meeting of the Governors at the hospital on Thursday, February 24th. Mr. Henry N. Custance presided, and among the governors present were Sir Arthur T. Watson, Bart., Q.C , Colonel the Hon. H. F. Eaton, the Hon. G. W. Spencer Lyttelton, Mr. G. B. Hudson, M.P., Mr. G. T. Marjoribanks (treasurer), Dr. Coupland, Dr. Pringle, and Dr. W. Pasteur. The Chairman was able to congratulate the Governors on the accounts of the year. The total income from all sources was £42,145, including legacies to the general and cancer funds amounting to £19,250; and the total expenditure amounted to £30,758. The ordinary income amounted to £19,920, against £20,055 in 1896, and the ordinary expenditure to £30,232. The board express their regret at the decline in the donations to the general funds, but attribute it to the Jubilee efforts and the diversion of half a-million to the Indian Famine Fund. On the other hand, the hospital received from the Prince of Wales's Fund £2,925. A donation of £1,000 from "Cambridge" towards the cost of the new cancer wing has an interesting story attached to it. First of all, £100 was sent in anonymously in an envelope with the intimation that there was more to follow, and further amounts came in in the same way until £1,000 was reached. The Chairman on behalf of the meeting expressed their thanks to the anonymous and mysterious donor for his handsome donation. The number of patients treated was larger than in any previous year. The total rose from 48,082 in 1896 to 50,893, but while the number of in-patients increased only from 3,598 to 3,641, or little more than 1 per cent., the out-patients show an increase in number from 44,484 to 47,252, or 6 per cent. This is mainly attributable to the casualty cases, and the question as to whether the number of these cases can properly be diminished and the expediency of appointing an inquiry officer is receiving the attention of the board. The average stay of surgical patients shows a slight diminution—from 30 92 days to 30·84 days—and in the case of the medical patients from 26·26 to 23·75 days. This is accounted for by the increased number of cases sent to the convalescent home at Clacton-on-Sea. With regard to this home, it is pointed out that, though it had to be closed against the admission of patients for several weeks, owing to the absence of a sufficient water supply, no less than 675 patients, in addition to 13 nurses from the hospital who required rest, were provided with accommodation. The works in connection with the new cancer wing of the hospital are being rapidly pushed on, arrangements have been made to remove the laundry to a more commodious position, and the work of erecting the new medical school buildings at a cost of £11,647 has been commenced. The loss by death of Dr. Henry Thompson and Mr. Leopold Hudson is referred to in the report. Mr. Hudson's memory will be perpetuated in the hospital through the generous action of his widow, who has presented a sum of £500 for the erection of a memorial window and tablet in the chapel and for the endowment of an annual prize for the student who passes the best examination in certain surgical subjects. The meeting unanimously re-elected the treasurer, the retiring members of the weekly board, the auditors, and other officers.

### CENTRAL LONDON OPHTHALMIC HOSPITAL.—ANNUAL MEETING.

The annual meeting of the Central London Ophthalmic Hospital, Gray's Inn Road, was held in the board-room of the hospital on Thursday, February 24th, 1898. Mr. Henry Clarke, in moving the adoption of the report, expressed his regret at the loss of their patron, the Duchess of Teck. He then dwelt on the growth of the work of the hospital and the manner in which the suggestion of the medical staff had been met for its enlargement by the purchase of the lease of the two adjoining houses. The additional property which has been acquired on favourable terms from Lord Calthorpe, and fourteen new beds, a dark room, and other conveniences, will be ready for use in a few weeks. The new secretary has justified his appointment, and has brought the finances of the hospital into excellent order; the committee of management have, therefore, been able to pay their way, and also to make preparations for meeting the needs of the neighbourhood. There is also a nice little nest egg in hand for the building fund, amounting, with the £1,219 paid for the lease of the extension, to £3,545. The ordinary income has been £1,389, and the expenditure £1,345. The board look forward to the visit of the committee of the Prince of Wales's Fund. It is thought that it will enable experts to judge the value of the work done at the hospital, and in the opinion of Mr. T. Britten Archer this would tend to remove the prejudice from which special hospitals still suffer in some quarters. The usual votes of thanks closed the proceedings.

### GREAT NORTHERN CENTRAL HOSPITAL.—ANNUAL MEETING.

The annual meeting of the Great Northern Central Hospital, Holloway Road, N., was held in the board-room of the hospital on Friday, February 25th, 1898, at five p.m. Mr. Murdoch took the chair, and read a most satisfactory report to a representative gathering of governors and subscribers. Stress was laid by all the speakers on the great need for extra accommodation and the advantages that would accrue to the hospital and to those using it if one of the wards unopened yet, though built four years ago, was occupied. In the first place, it is impossible to admit many patients that ought to be taken in; and, in the next, convalescents were turned out at the earliest possible moment—a state of things most undesirable. The Chairman showed that the expenses of the administration would not be increased by opening the new ward, and that would bring down the average cost per bed to £50 per annum, instead of £70, as at present. Consequently, they would compare favourably with other hospitals, and the number of beds then being over 100, might possibly secure them a larger grant from the Prince of Wales's Fund. The hospital has not joined the Central Hospitals Board, for the reason that they are really the instigators of the scheme of systematic inquiry into the circumstances of the out-door patients now advocated by others. It has been by them worked successfully for some years. The festival dinner, thanks to the efforts of its chairman (Mr. Cohen, M.P.), resulted in donations amounting to £3,746. The inhabitants of Highgate, and round about it, to quote the words of Mr. Story, who represents the working men on the Board, wanted to enjoy themselves, to celebrate the Diamond Jubilee and do the hospital a good turn at the same time, so that the sum of £1,000 was raised and a bed endowed, of which the working men contributed £105. One gratifying feature of the year's finance is the increase of annual subscribers, another is that the income amounted to £15,747 and the expenditure to £10,257, the balance of receipts over expenses, namely, £5,500, being invested. Notwithstanding the handsome surplus, the fact that the hospital is £9,000 in debt for maintenance is not lost sight of by the committee, and efforts will be made towards clearing it off. The greater portion of the surplus this year, being due to extraordinary receipts, and being, for the most part, moneys for endowment, has been invested. Still it is only fair to note that the hospital buildings and furniture are debt free and that every source of income, excepting that from legacies, has increased. The uniform system of accounts has been adopted. The report was moved by the chairman (Mr. Murdoch), seconded by Mr. Burdett Coutts (hon. treasurer), and unanimously carried. The usual votes of thanks were put and carried: one to the Ladies' Association Endowment Fund brought into prominence the names of the Baroness Burdett Coutts, Mrs. Murdoch, Mrs. Harkness, and Mrs. Furley. One-third of the committee of management retired and were re-elected for a term of three years. The hon. officers were reappointed, and Mr. J. B. Braithwaite was elected vice-president. Dr. W. A. Malcolm and Mr. Walter Reynolds, J.P., were elected to the committee of management. Mr. H. W. Allingham was congratulated on his appointment as surgeon-extraordinary to the Prince of Wales.

### ST. JOHN'S HOSPITAL FOR DISEASES OF THE SKIN.

Lord Chesterfield presided over the thirty-fourth annual general board of governors of this hospital held at the Hotel Victoria on Friday, February 25th. The report showed that during last year the in-patients admitted numbered 270, against 247 in 1896, the average duration of treatment being 44 days, against 36 days. The total number of new out-patients was 6,898, against 6,928. In consequence of pressure on the in-patient department the number of beds has been increased from 40 to 50, and an isolated cottage at the rear of the hospital provides seven beds for contagious diseases. With regard to the finances of the institution, the income amounted to £4,435, and the expenditure to £4,396. For the first time the annual subscriptions reached four figures, namely, £1,118, against £896, and the donations, amounting to £731, also show an increase of £116. The income from in-patients amounted to £256, and from out-patients £2,134. The financial position of the hospital was referred to in glowing terms by the Chairman, who observed that the surest way of making a charity successful is to show that it is well managed. For 1897 there was a large decrease in their management expenses. Every item had been reduced, and while in 1896 the total was £661, last year it amounted to £433. In 1896 maintenance cost £4,095, in 1897 £3,634, while the cost per bed had been reduced from £104 to £80. The fact that the in-patient and out-patient departments were carried on in different parts of London added to the difficulties of management, but they hoped some day to have them both under one roof. Mr. Van Praagh, in seconding the adoption of the report, remarked that those who had formerly criticised the hospital were now the most ready to accord it praise. The motion was unanimously carried. In responding to a vote of thanks to the medical staff Dr. T. D. Savill said that in other European cities skin hospitals were provided with a small laboratory and museum in order that microbes might be studied, and it was a matter for consideration whether this example should not be followed here.

### SEAMEN'S HOSPITAL SOCIETY, "DREADNOUGHT."

The Bishop of London presided over the annual meeting held at the United Service Institution on Friday, February 25th. There was a very large attendance, including Colonel Milward, M.P., the Rev. Brooke Lambert, Admiral Coote, C.B., Commander Augusto Bianco (Italian Naval Attaché), and Mr. G. Lidgett. In an eloquent speech the chairman commended this charity to all men of British race. We owed, he said, our national greatness in a large measure to our seamen, who had claims on us we could not resist. The hospital provided for sailors of every nationality, so that its claims were of a universal character. The report shows that the Jubilee year was an exceptionally prosperous one for the society, which, instead of showing a deficiency, as it has done for years previous, has a balance on the right side. Touching on this point Colonel Milward, in moving the adoption of the report, observed that charities sometimes liked to hide the fact that their income exceeded their expenditure. He considered, however, that this was a bad plan, and he believed the public had more confidence in the management of an institution that paid its way. The total income amounted to £22,254, and the expenditure to £17,768. The sum of £3,000 has been invested in Consols, and a balance of £1,485 carried forward. The Prince of Wales's Fund contributed £1,418, and from American citizens in London a sum of £1,000 was received for the endowment of a bed in the Branch Hospital. The number of patients under treatment at the two hospitals and two dispensaries of the society during 1897 was 25,201. At the "Dreadnought" Hospital 2,167 in-patients and 8,801 out-patients were treated, while at the Branch Hospital the in-patients numbered 453 and the out-patients 9,918. At the East India Dock Road Dispensary and the Gravesend Dispensary respectively 3,628 and 954 out-patients were treated. It is pointed out that not only has the number of patients greatly exceeded that of any previous year, but the total is three times as large as the total ten years ago, and nearly twice as large as the average of the last ten years. The result of this is that the committee have to face an increase of expenditure. A large portion of this increase is caused by the extended sphere of usefulness of the Branch Hospital. To make the proposed enlargement here effectual it will be necessary to add 20 beds and a new out-patient department at a cost of upwards of £8,000. The architect has prepared plans which have been approved by the committee, and they only await the necessary funds to proceed with the work. It was hoped that the Admiralty might assist in re-flooring the wards at Greenwich, which have become rough and absorbent from wear. The committee, however, have received intimation that their request cannot be entertained. The cost of re-flooring the wards will be about £1,900, and it is felt that this work or a portion of it will have to be taken in hand before long. It is interesting to note that of the 2,620 in-patients treated, 1,737 were natives of England, Scotland, Ireland, and Wales, while 227 were East Indians, 105 Swedes, 100 Norwegians, 29 Chinese, 20 Japanese, 71 Germans, and 32 Danes. The officers having been elected, cordial votes of thanks were tendered to the numerous contributors to the funds, and to the Bishop of London for presiding.

### THE CITY OF LONDON HOSPITAL FOR DISEASES OF THE CHEST.

The annual general meeting, held at Gresham College on Monday, February 28th, was presided over by Mr. Joseph Benson. The past year has not been a satisfactory one for this institution. In June last, owing to want of funds, the committee were compelled to close upwards of 120 beds, and though the financial position improved later in the year, there are still 100 beds closed out of 164 the hospital contains. It was stated by the Chairman that there are a large number of patients constantly waiting for admission, and that the crippled condition of the hospital is a serious calamity for the East-end of London. The in-patients during the year numbered 760, a large falling off compared with 1896; while the out-patients reached 15,829, the total number of attendances being 65,126. The hospital's total income for 1897 amounted to £9,380, and the expenditure to £9,225. At the close of 1897 the loan from the bankers amounted to £3,000, but the amount has since been wiped off by the realization of stock. The investments held by the hospital have consequently been reduced to £2,666. From the Prince of Wales's Fund the sum of £927 has been received, and this amount will be included in the accounts of the current year. A special appeal for funds was sent out just before Christmas, and up to the present it has produced about £1,000. Owing partly to the action of the Committee of the Hospital Sunday Fund a sub-committee has been appointed with a view of deciding as to the desirability of appointing an officer who should be empowered to inquire into the pecuniary position of persons seeking relief, especially in the out-patient department. With regard to the management of the hospital it is noteworthy that Sir Edward Sassoon, Bart., Sir M. Bhownaggree, M.P., Mr. Alderman Truscott, Major Coates, Mr. Andrew Motion, Mr. Worthington Evans, and Mr. Frank Hyland have been added to the committee with a view of strengthening that body, and that at Monday's meeting Sir Edward Sassoon, on the motion of Sir M. Bhownaggree, was elected treasurer in place of Mr. Julian Hill, who has resigned after many years' service.

### ROYAL HOSPITAL FOR CHILDREN AND WOMEN.

The Lord Mayor presided over the eighty-first general court of the governors of the institution held at the Mansion House on Monday, February 28th. The reports presented would have been of a more favourable character but for a case of diphtheria, which necessitated the closing of the hospital for three months for disinfection, painting, and construction of new sewers and drains. The work cost over £1,000, and as a result the committee are indebted to their bankers for the sum of £1,030. Owing to the closing of the hospital the in-patients for the year numbered only 375, against 528 in 1896, and the out-patients 6,525, against 7,811. The statement of accounts shows that the ordinary income amounted to £4,221, and the ordinary expenditure to £3,041. The extraordinary income amounted to £518, while the extraordinary expenditure consisted of £731 for special repairs, and £968 legacies invested. The liabilities at the end of the year amounted to £1,288, £1,030 being due to the bankers, £198 to tradesmen, and £60 for rent, rates, and taxes. The Lord Mayor moved that the report be adopted, and "That the Royal Hospital for Children and Women is in every way worthy of support, and has strong and peculiar claims upon the City of London; and that a subscription list be opened at once to free it from debt." His Lordship, in speaking to the resolution, said he knew of no institution more worthy of support, and expressed his intention of visiting the hospital if he could possibly spare the time. The resolution having been carried, the officers were re-elected, and a vote of thanks given to the medical staff. In replying to this, Dr. Haig said that in some of the hospitals abuses were alleged to exist in the working of the out-patient department. But so far as this hospital was concerned, there was as little abuse as in any in London, and for that reason the medical staff were delighted to work for it. A vote of thanks to the Lord Mayor and Lady Mayoress concluded the proceedings.

### KING'S COLLEGE HOSPITAL.

Mr. Richard Twining presided at the annual court of governors of King's College Hospital, held at the hospital on Thursday, February 24th. Among others present were Viscount Dillon, Rev. D. Robertson, Hon. Conrad Dillon, and Rev. N. Bromley (warden). The report indicates that during the year the total number of in-patients under treatment was 2,604, the daily average of beds occupied being 178. In the out-patient department the new cases were 23,877, of which 13,143 were accidents, and 633 poor women received attention during confinement in their own homes. With regard to the alleged abuse of the out-patient departments of the London hospitals, the report states that in this hospital the claims of the out-patients continue to be carefully considered by an out-patient registrar, and the committee are still of opinion that the "abuse," if any exists at King's College, is very small, and that more harm than good would be done by any system more inquisitorial than the present, which has been found to work well, and without giving unnecessary pain to the deserving poor. The accounts show that the income during the year, including legacies of £6,219, amounted to £21,205. The ordinary expenditure amounted to £17,885, and the extraordinary expenditure to £780. The report having been adopted, a warm tribute was paid to Mr. Richard Twining for his services to the hospital as treasurer, a post which he now resigns. Mr. Charles Awdry was elected to the position vacated by Mr. Twining, and the meeting closed with the usual votes of thanks.

### NATIONAL ORTHOPÆDIC HOSPITAL.

His Grace the Duke of Marlborough presided on Monday at the annual meeting of the National Orthopædic Hospital in Great Portland Street. The report of the Committee of Management, read by the Secretary, stated that during the year ended December 31st, 1897, 123 children and 83 adults had been received as in-patients, the average period of residence being 85 days, while 1,279 out-patients had paid 3,124 visits to the hospital. At the request of the medical staff the committee had arranged at considerable expense for an isolation ward, so that any case where there was a suspicion of infection might be at once removed from the general wards. The constant care exercised by the medical staff, the entirely honorary character of which was emphasised by the report, had been specially exemplified by the fact that the senior surgeon, Dr. Little, had, at the risk of his life, sucked the poison from the throat of a diphtheric patient, thereby contracting a very serious illness. The ladies' committee had again been of the greatest service. The sale of work for 1897, opened by the Duchess of

Marlborough, had realised £132 9s. 6d. During the year £73 had been paid in aid of patients, and the ladies had ministered in many ways to their comfort. The greatest work of the Ladies' Committee for the year had been to provide the total cost of the upper balcony in Great Portland Street, so that the children in the Robert Viger's ward might have like privileges with those of the Samuel Fielden's ward. The donations, which for several years had been growing in volume, had this year shown a decrease of £200, consequent, it was felt, upon the overshadowing influence of the Prince of Wales's Fund. The total income of the year amounted to £2,150, and the expenditure to £2,278, leaving a balance of £128 on the wrong side in the accounts. The sum of £70 was received from the Prince of Wales's Fund, but in sending this donation the secretary stated that it would not be repeated. On this point the report declares that "the adoption of the rule that no hospital having less than 100 beds is to share in the income of the Fund, deprives us of any hope we had of further participation in the Fund." Mr. Cooper, however, in addressing the meeting, said that the compulsory rebuilding of the Bolsover Street wing would provide an additional 30 or 40 beds. Lord Farquhar moved that the retiring members of the Committee of Management be re-elected, and this and other formal resolutions were unanimously adopted.

